# Awareness and knowledge of interventional radiology among medical students at an Indian institution

**DOI:** 10.1186/s42155-019-0093-x

**Published:** 2019-12-27

**Authors:** Deepsha Agrawal, Michael Alan Renfrew, Sulove Singhal, Yash Bhansali

**Affiliations:** 1 County Durham and Darlington NHS Foundation Trust, Darlington, UK; 2 College of Science and Engineering, University of Edinburgh, Edinburgh, UK; 3 Pt Jawaharlal Nehru Memorial Medical College, Raipur, India; 4 Syracuse University, New York, USA

**Keywords:** Interventional radiology, Medical education, Undergraduate curriculum, Medical schools, Interventional radiology knowledge, Interventional radiology awareness, Interventional radiology career

## Abstract

**Purpose:**

Interventional radiology (IR) is a novel and evolving sub-specialty that encompasses image guided diagnostic and therapeutic procedures. With the advent of new imaging techniques and an increasing demand of minimally invasive procedures, IR continues to grow as a core component in medical and surgical therapeutics. Radiology teaching is a part of medical undergraduate curriculum; however, the medical undergraduate cohort lacks exposure to IR principles, methods and techniques. The purpose of this study is to determine the knowledge and awareness of IR among medical students in a single university in India.

**Materials and methods:**

Electronic anonymous surveys were sent to 350 medical students of Pt. JNM Medical College, Raipur, India. Each survey comprised of questions assessing knowledge and exposure to IR. A total of 70 students (20%) responded.

**Results:**

85.7% of respondents positively reported that radiologists have a role in diagnostic as well as therapeutic interventions, however, 60% of students cited a very poor/poor knowledge of IR. A larger part, 91.5%, stated that they would be interested in IR based teaching delivered as a part of their undergraduate teaching program. Those who knew at least one interventional radiology technique were 1.51 (95% CI: 1.02–2.22; *p* < 0.05) times more likely to be considering it as a career.

**Conclusion:**

Medical Students demonstrate a poor knowledge of IR. This corresponds to a limited and inconsistent exposure to IR in medical schools. The study suggests that there is a need to deliver an IR based curriculum in medical undergraduate teaching in India. Our proposition includes introducing a regulated IR teaching in undergraduate medical education using new module designs and presenting medical students an opportunity to attend IR education days, symposiums and conferences to incite early participation.

## Background

**Fig. 1:**
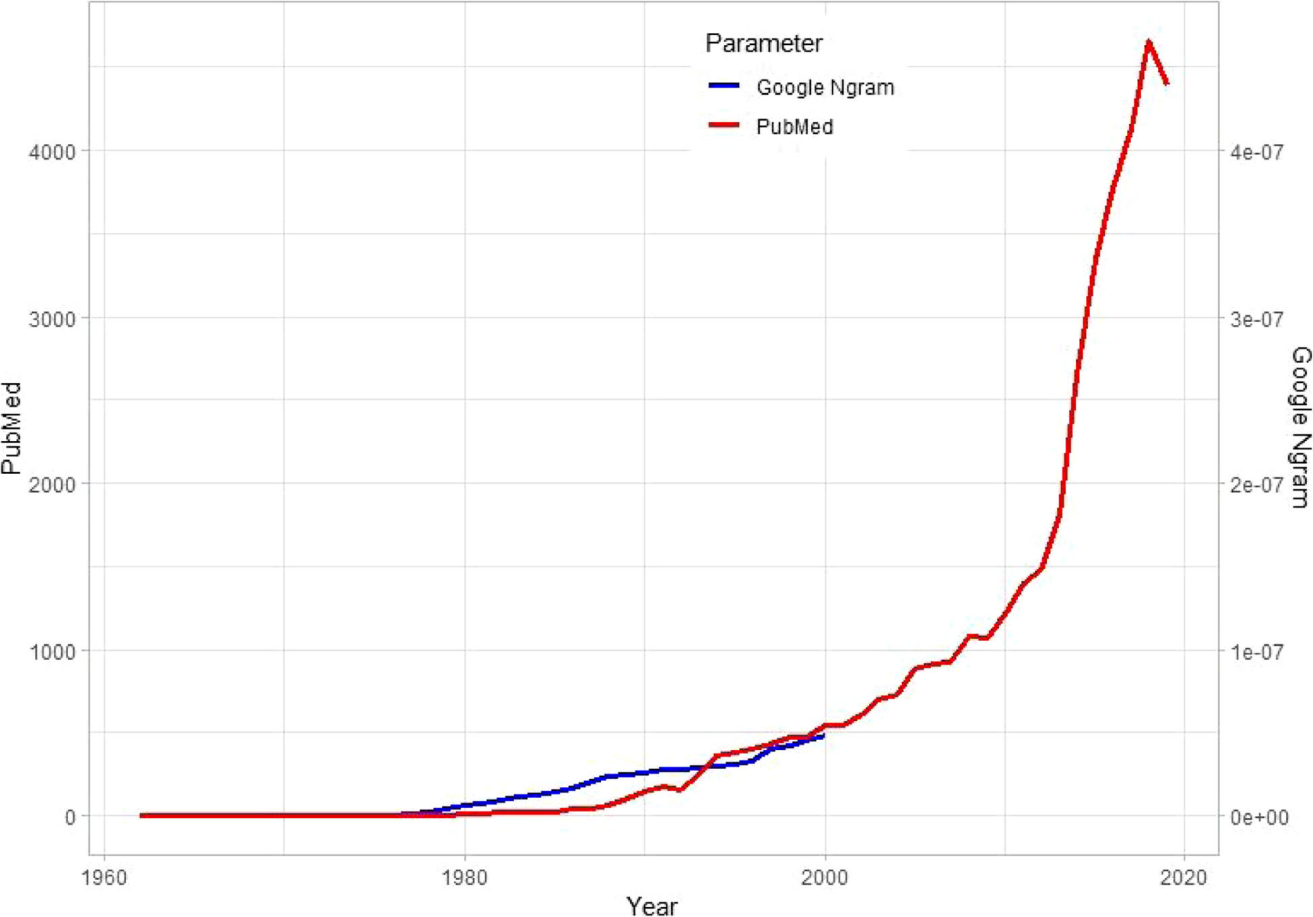
A graphical representation of texts indexed under word string ‘interventional radiology’ on Google English corpora and PubMed between 1960 and 2019. There is an exponential rise in quotation of ‘interventional radiology’ on PubMed and indexed compilation on Google

Interventional radiology (IR) has experienced an unprecedented growth in recent years. Having evolved from limb saving angioplasties in 1964 to over 50 therapeutic procedures today, the subspecialty of IR continues to flourish. A look at the Google ngram for phrase ‘interventional radiology’ aptly and interestingly represents the flourishing practice of IR. This graph charts the frequency of ‘interventional radiology’ search string in Google’s text corpora in English. It begins at a frequency of 0.0000000092%, being at the baseline of 0.00000000% until then, and exponentially rises up to a frequency of 0.0000057220% in 2008 (Fig. 1).

A key World Health Organization (WHO) report entitled ‘Efficacy and radiation safety in interventional radiology’, published in 2000, also concluded that IR has a growing scope of practice in treating diseases of cardiovascular and non-vascular origin in both developed and developing countries (Worldcat.org 2019). However, due to only recent accreditation of IR as a sub-specialty, modules for IR based teaching have not been amalgamated into undergraduate medical curriculum. In several studies across different countries, medical students have demonstrated a poor understanding of IR, owing to suboptimal teaching and exposure levels (O'Malley and Athreya 2012; Atiiga et al. 2017; de Gregorio et al. 2018; Commander et al. 2014; Leong et al. 2009; Ghatan et al. 2010; Nissim et al. 2013; Foo et al. 2018; Ojha et al. 2017).

As technical means to provide minimally invasive interventions with better outcomes continue to grow, it is of paramount importance to train the future workforce. The Cardiovascular and Interventional Society of Europe (CIRSE) (Cirse.org 2019), British Society of Interventional Radiology (BSIR) (Bsir.org 2019) and the Society of Interventional Radiology (SIR) (Sirweb.org 2019), and they have been continuously working towards addressing a lack of IR knowledge and awareness among medical students.

There is a lack of formal evidence from India, however, there have been reports of a steady growth in technology and therapeutic radiology procedures. This study is derived from a survey presented to second, pre-final and final year medical students in an Indian university. It was conducted to appraise the current understanding and perception of IR among medical students. The results represent the current awareness and understanding of IR among medical students under the present undergraduate teaching curriculum.

## Material and methods

An electronic survey with 12 questions was designed based on the studies conducted by Gregorio et al. (2018) and Leong et al. (2009). This electronic survey was distributed among 350 medical students of Pt. Jawarharlal Nehru Memorial Medical College, Raipur, India. The survey was entirely electronic, anonymous and participation was voluntary. Responses were collected during July 2019, starting from 1 July 2019 to 31 July 2019.

The questions demanded a mandatory response on year of medical school. All students from the first year of medical school and internship were excluded from the study. Questions were based on a single choice between ‘Yes’ and ‘No’. Some questions took into account the level of knowledge/awareness and were marked on a range of very poor to very good or strongly disagree to strongly agree. Questions relating to understanding of procedures accepted multiple responses against designated procedures on the survey form. The form allowed students to enter an expansive answer regarding their concerns about radiation exposure in the practice of IR.

### Statistical analysis

Responses collected from the survey were collated in a spreadsheet program (Excel, Microsoft 2010) and analyzed using statistical software (R-3.6.1 for windows 10). Data were tested using Fisher’s exact test. *P* value was established at 0.038 (*P* < 0.05).

## Results

**Fig. 2:**
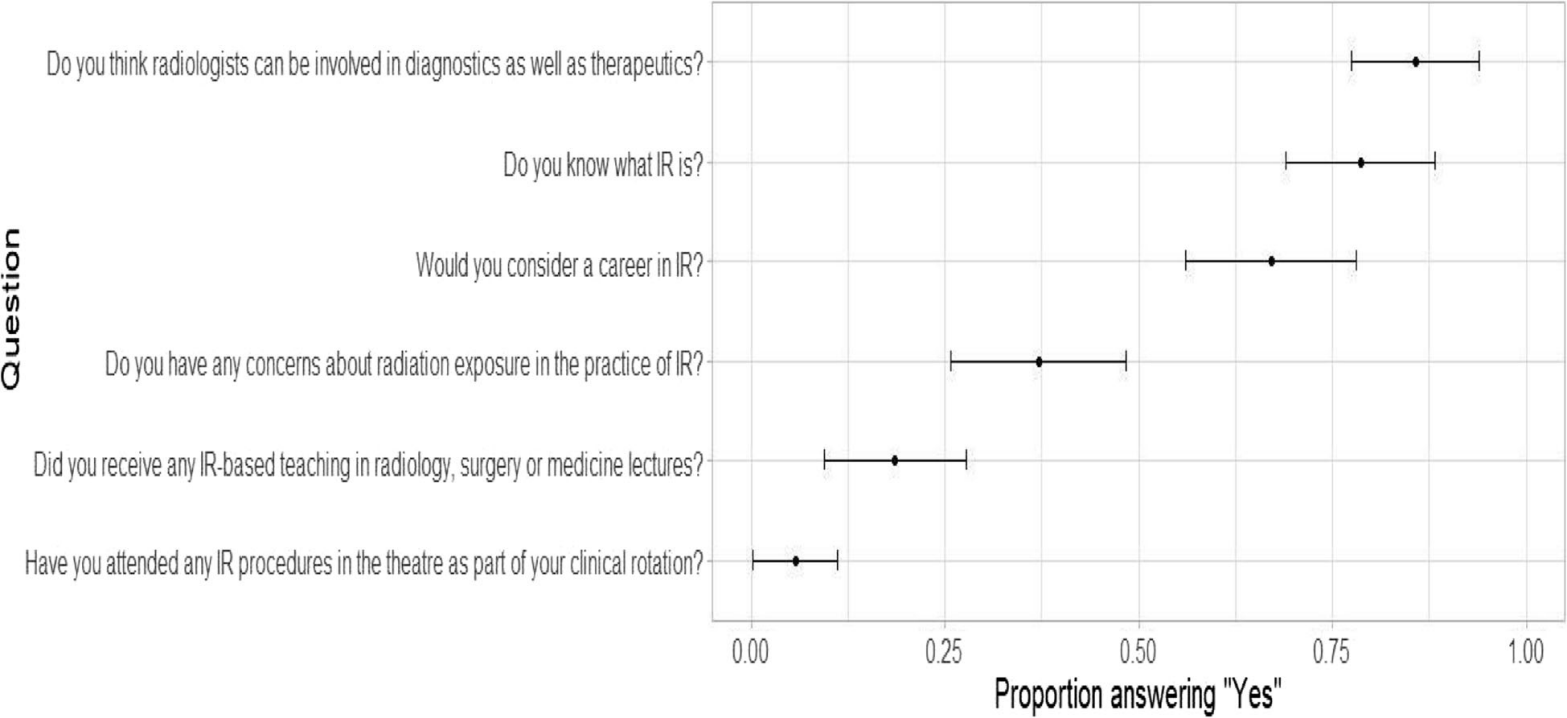
*N* = 70. An analysis of survey questions (y-axis) and responses (x-axis) depicted on a 95% CI. Responses to the survey questions indicate that about 70% of students know what IR is and would consider a career in the sub-specialty. Howbeit less than 25% students received an IR theory/ practical teaching

**Fig. 3:**
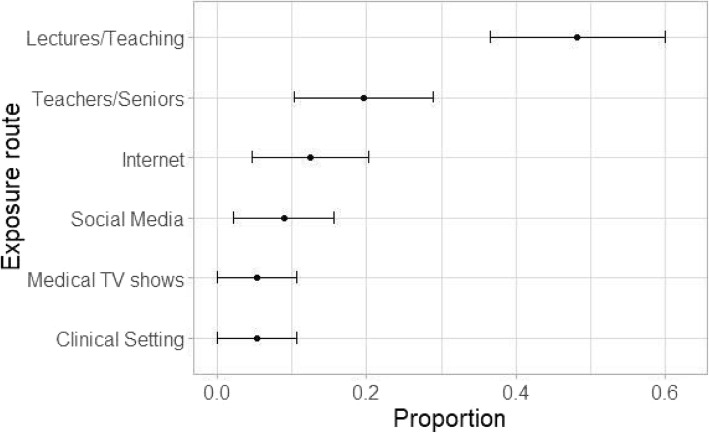
*N* = 59. Response of students to survey question ‘How were you first exposed to IR?’. Most students attribute awareness of IR through lectures/ teaching in medical schools

A total of 70 (20%) out of potential 350 students responded to the survey. These participants were distributed across pre-clinical and clinical year- 16/22.9% in pre-clinical (2nd year) and 54/77.1 in clinical (pre-final and final year). Most of the students- 85.7% knew that radiology could be diagnostic as well as therapeutic. However, the number slightly dipped down to 78.6% when asked whether they understand what IR is (Fig. 2). Of those who stated they discerned what is IR, the majority (49%) indicated that it was through classroom teaching. The remainder answered as follows: 20% through guidance by teachers/senior colleagues outside of formal classroom teaching; 12% via internet resources; 9% through social media; 5% by means of clinical setting and interestingly 5% through the medium of medical TV shows (Fig. 3).

**Fig. 4:**
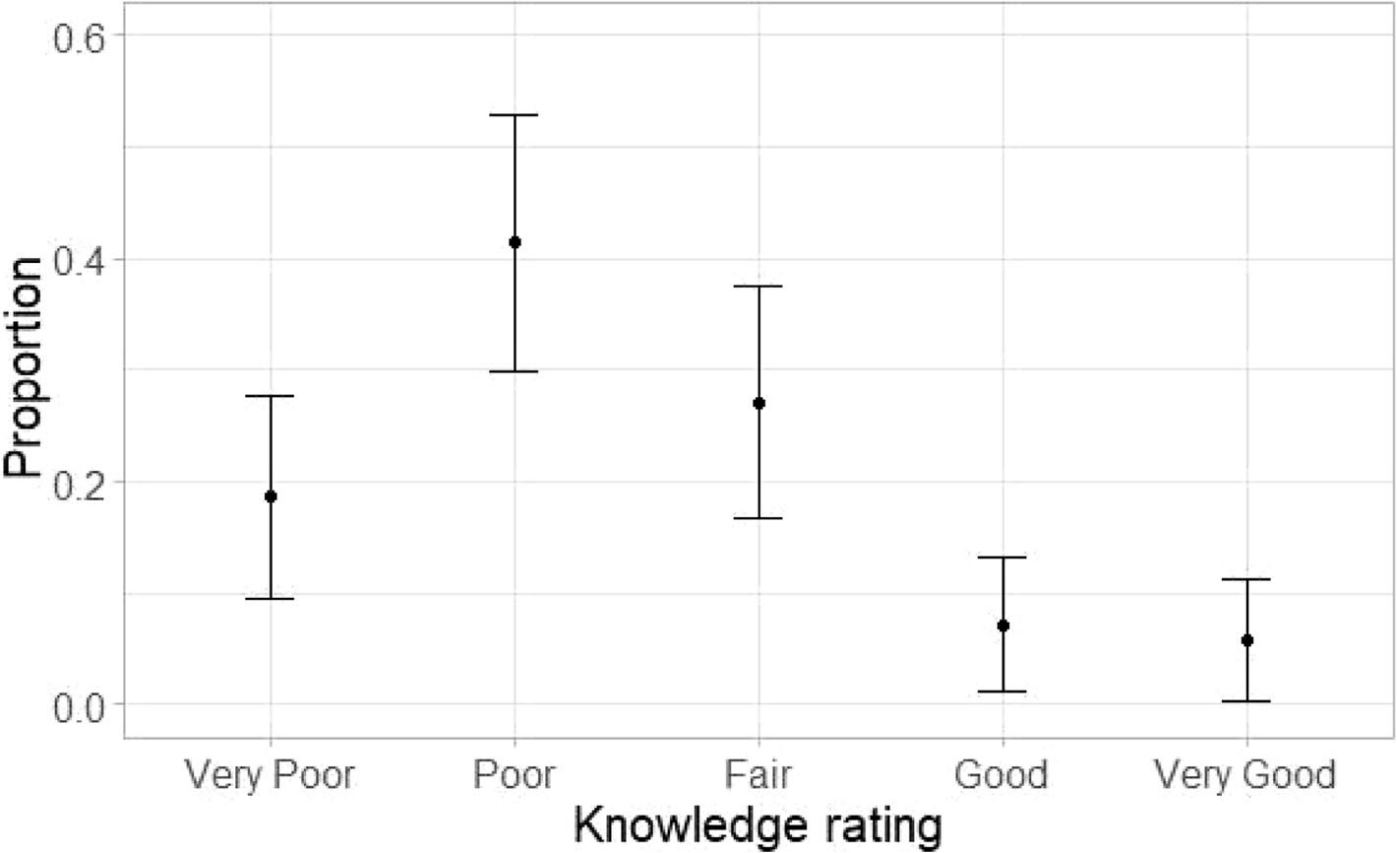
N = 70. Responses to survey question ‘How would you rate your knowledge of IR?’. A proportion of 0.41 and 0.18 estimate their IR knowledge as ‘poor’ and ‘very poor’ respectively

Upon being asked to self-evaluate the knowledge of IR as compared to other medical specialties, most students admitted poor to very poor knowledge. 41.4 and 18.6% students indicated poor and very poor knowledge, respectively against a 27.1% reporting a fair level of knowledge of IR. Only 7.1 and 5.7% students agreed to having good and very good knowledge, respectively (Fig. 4).

The next question surveyed if the students had received any introductory teaching on IR in medicine or surgery classes. A major percentage of students, 81.4%/57, refused any introductory teaching while a smaller percentage, 18.6%/13 answered that they did. Out of the 13 students who answered yes, 10 expressed where they received this introductory teaching. Most (70%) of this was indicated as teaching in dedicated radiology classes.

However, on being surveyed on the scope of IR procedures the respondents attended as part of their clinical rotation, 94.3% registered a ‘No’ as an answer.

The students were then surveyed on the perception of the kind of procedures interventional radiologists do in daily practice. A total of 7 options were presented out of which 5 were correct answers. The options included, endovascular coil embolization, appendectomy, ultrasound guided biopsies, balloon angioplasty, hysterectomy, uterine fibroid embolization and percutaneous nephrostomy. Over 41 % of students said they had no idea, about 6% answered incorrectly and 53% answered correctly choosing among the 7 options.

**Fig. 5:**
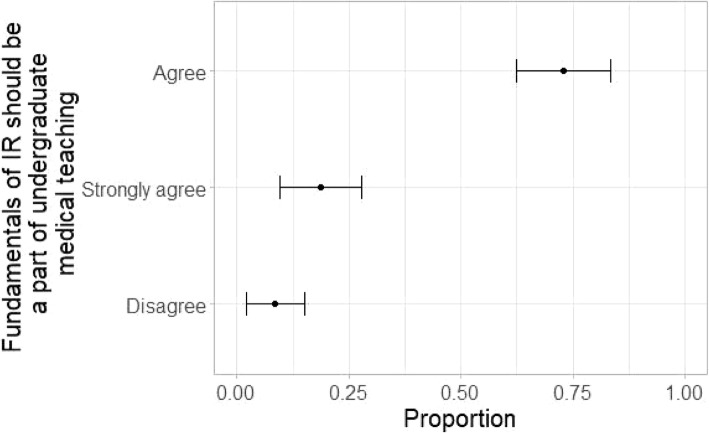
N = 70. Responses to survey question ‘Do you agree that basics of IR should be a part of medical undergraduate teaching?’. Majority of medical students agree to integrating IR in undergraduate medical teaching

As a concluding assessment to overall perception of medical students, it was asked if they feel there should be more IR-orientated teaching as a part of the undergraduate curriculum. More than two-thirds of students agreed to this proposition. Of the students who agreed, 72.9% marked agree and 18.6 marked strongly agree (Fig. 5).

The students also acknowledged an unaddressed concern regarding radiation exposure in IR with 62.9% of respondents disclosing that they were concerned, nonetheless 67.1.% said they would consider a career in IR.

## Discussion and conclusion

The most important finding of the study was that those who were familiar with at least one interventional radiology technique were 1.51 (95% CI: 1.02–2.22; *p* < 0.05) times more likely to be considering it as a career. These figures suggest that a knowledge and awareness of IR principles and techniques may precipitate an active interest in the field. However, it may be argued that the effects are correlated with students who are already interested in radiology making more effort to learn IR fundamentals. The other important finding is that the students expressed a lack of knowledge and insufficient exposure to IR in their undergraduate years.

Interventional radiology is one of the most dynamic fields in medicine and as it continues to evolve, there is a need to incorporate IR within the undergraduate curriculum appropriate for medical schools. A lack of IR as a discipline within undergraduate teaching modules could have a direct impact on both, the choice of IR as a career and an understanding of treatment options available to patients. This, in turn, suggests a need to revise the current radiology curriculum and clinical rotations to introduce IR at an early stage of medical education.

It is recommended that medical students receive a curriculum-based teaching and the learning goals be focused on common acute clinical problems managed with image-guided interventional treatments (Table 1). Enriching the undergraduate cohort with the fundamental principles underpinning IR may further a critical understanding of it (Shaikh et al. 2015; Alexander et al. 2015; Lee and Lee 2017; Goldman et al. 2018). This curriculum would ideally be amalgamated into medical school and internship assessments.

**Table 1:** Principle IR subjects, presently

Condition	Treatment
Peripheral Arterial Disease	Angioplasty and stenting
Aneurysm	Endovascular Repair (EVAR)
Venous Thromboembolic Disease	Catheter-directed thrombolysis, balloon angioplasty, or stenting. Alternatively, IVC filter
Uterine Fibroids	Uterine artery embolization
Benign prostate hyperplasia (BPH)	Prostate artery embolization
Biopsies and drainage	Image-guided access using US, CT or fluoroscopy
Vascular Malformations	Sclerotherapy
Interventional Oncology	Radiofrequency ablation, cryoablation, chemoembolization
Stroke	Intra-arterial thrombectomy treatment
Portal Hypertension	Transjugular intrahepatic portosystemic shunt (TIPS) procedure

The cardiovascular and interventional society of Europe (CIRSE) recommends that the medical students IR curriculum should deliver the following (Cirse.org 2019):

- The basis of interventional radiology and its historical context.
- Image guidance for interventional procedures.
- Knowledge of radiation protection guidelines for interventional procedures.
- Knowledge of the legislation relating to the use of interventional radiology in.
- clinical practice.

Similar studies have been done in Europe which demonstrated a suboptimal exposure of IR among medical students. In recognition of the existing issue among medical students in European countries and the importance of education at an early stage, the CIRSE has published a curriculum of interventional radiology for medical students that provides an extensive guidance on IR learning goals in medical schools.

There are other ongoing efforts to address this shortfall. The Be InspIRed initiative by CIRSE works to promote the IR educational model for medical students by supporting attendance at scientific sessions and simulation sessions. With a participation of over fifteen hundred students over the last few years, this has proved to be an effective way of generating interest in the field. Going forward, the CIRSE also recently introduced the European Trainee Forum (ETF) which concentrates its efforts on introducing and expanding educational opportunities for trainees and residents. The success of these programs emphasizes the importance of promoting early interest in IR as a promising strategy to address the lack of IR knowledge among students.

The present study has several limitations. Firstly, a relatively small number of students were surveyed. Secondly, these students belonged to the same university, therefore had similar academic environment. It was also noted that many respondents indicated IR teaching by private tutors. as an education tool. This is certainly a valuable knowledge shift, however, is not delivered as a part of undergraduate medical curriculum. Therefore, further studies are needed to validate our proposition.

In conclusion, this study demonstrates that medical undergraduate students in India have a poor understanding of IR due to limited exposure to the sub-specialty. An intervention, in the form of introducing IR in medical undergraduate teaching may have a positive impact on the knowledge and skills of medical students and a critical understanding of the scope of practice in IR.

It is acknowledged that the inception of these developments will be a challenge; particularly facets such as advancing these ideas to regulatory bodies and thereafter working in partnership with them to develop and deliver a new curriculum.

## Data Availability

The datasets used and/or analyzed during the current study are available from the corresponding author on reasonable request.

## Abbreviations

BSIR: British Society of Interventional Radiology
CIRSE: Cardiovascular and Interventional Society of Europe
IR: Interventional Radiology
SIR: Society of Interventional Radiology
WHO: World Health Organization
